# New-onset diabetes mellitus in patients with COVID19 infection admitted to a tertiary care hospital: A single-center experience

**DOI:** 10.12669/pjms.40.8.8797

**Published:** 2024-09

**Authors:** Muhammad Abdur Rahman Afridi, Zafar Ali, Naveed Iqbal, Usman Zeb

**Affiliations:** 1 Muhammad Abdur Rahman-Afridi, FCPS Professor of Medicine Department of Medicine, Lady Reading Hospital, Medical Teaching Institution, Peshawar, Khyber Pakhtunkhwa, Pakistan; 2 Zafar Ali, FCPS Department of Medicine, Lady Reading Hospital, Medical Teaching Institution, Peshawar, Khyber Pakhtunkhwa, Pakistan; 3 Naveed Iqbal, FCPS Department of Medicine, Lady Reading Hospital, Medical Teaching Institution, Peshawar, Khyber Pakhtunkhwa, Pakistan; 4 Usman Zeb, Department of Medicine, Lady Reading Hospital, Medical Teaching Institution, Peshawar, Khyber Pakhtunkhwa, Pakistan

**Keywords:** COVID-19, New-onset Diabetes mellitus, NODM, Sars-Cov-2

## Abstract

**Objective:**

To determine the frequency of new-onset diabetes mellitus (NODM) in patients with COVID-19 in a tertiary care hospital.

**Method:**

It was a retrospective descriptive study carried out in Lady Reading Hospital Peshawar, Khyber Pakhtunkhwa province of Pakistan from November 2021 to April 2022. All patients having new onset Diabetes Mellitus (NODM) were identified among a total of 300 patients admitted to the hospital with COVID-19 infection. Patients’ data including relevant investigations were accessed through the hospital management information system (HMIS). SPSS version-23 was used for data entry and statistical analysis.

**Results:**

Out of 300 COVID-19 patients included in the study, 163 (54.3%) were female and 137(45.7%) were male. The mean age of the patients was 56.80±13.72 (IQR 15) years. Frequency of the new onset diabetes was 44(14.7%); 19 (6.33%) male and 25(8.33%) female. Among the 44 NODM patients, the majority (57%) were female (*p*=0.720). Most (64%) of the patients with new-onset DM were in the middle age (*p*=0.018).

**Conclusion:**

A significant number of patients with COVID-19 infection are prone to develop new-onset diabetes during their admission to the hospital.

## INTRODUCTION

Coronavirus disease (COVID-19) is caused by severe acute respiratory syndrome corona-virus 2 (SARS-CoV-2), a novel coronavirus that was first identified in Wuhan city of China, in December 2019. The rapidly emerging COVID-19 pandemic has caused major public health challenges globally with an average mortality rate of 6%, ranging from 1-14.4%.^1^ Diabetes is associated with an increased risk of COVID-19 severity and poor outcomes^2^, including more severe respiratory involvement, ICU admissions, need for mechanical ventilation, and increased mortality. A bidirectional relationship between DM and COVID-19 has been suggested.^3^

Observational studies and case reports suspected the diabetogenic effect of COVID-19 infection based on the findings of new onset of diabetes, worsening of glycemic control in pre-existing diabetes, and occurrence of severe metabolic complications in previously known diabetic patients.^2^,^4^,^5^ The development of new-onset diabetes in COVID-19 may be due to the direct pancreatic beta cell damage by the virus entry, the body’s stress response to infection including cytokine storm, and the use of diabetogenic drugs such as corticosteroids in the treatment of COVID19.^3^,^6^ A study from Wuhan, China, reported 20.8% newly diagnosed diabetes mellitus based on initial glucose measurement upon admission.^7^

A meta-analysis of 3,711 COVID-19 patients from eight studies including 492 NODM patients, the pooled prevalence of NODM was 14.4%.^8^ Another systematic review of 27 studies revealed NODM in 16.1% patients. Of these, 9.74% reported in the developed world and 19.5% in the developing world.^9^ The risk of mortality appears to be higher in patients who develop new-onset diabetes during COVID-19 infection than those who are already diabetic.^7^,^10^

Based on the fact of increased mortality associated with new-onset diabetes mellitus in patients with COVID-19, we felt the need to study it locally. We, therefore, designed this study to determine the frequency of new-onset diabetes mellitus in patients with COVID-19 presenting to a tertiary care hospital. The findings of this study will help in the early detection and diagnosis of NODM. Consequently, early institution of treatment strategies directed at better glycemic control and vigilance regarding the detection of severe metabolic complications of diabetes mellitus, like diabetic ketoacidosis (DKA), will be emphasized. This will ultimately help in the reduction of morbidity, mortality, and burden on COVID-specific wards and high dependency units (HDUs) in the hospitals. **The objective was** to determine the frequency of new-onset diabetes mellitus in patients with COVID-19 presenting to a tertiary care hospital.

## METHODS

**This r**etrospective observational study **was carried out in the** Department of Medicine/COVID Complex, Lady Reading Hospital (LRH) Peshawar, from November 2021 to April 2022. Data of 300 COVID-19 patients were included, keeping 20.8% prevalence^7^ of the new-onset diabetes mellitus in patients with COVID-19; 95% confidence interval, and 5% margin of error.

### Operational definitions:

### COVID-19:

Patients with acute respiratory symptoms like fever, cough, and shortness of breath; and radiological findings of pulmonary infiltrates; and positive Antigen based rapid detection test, and Reverse transcription polymerase chain reaction (RT-PCR) on nasopharyngeal swabs were considered as COVID-19.

### New-Onset Diabetes Mellitus (NODM):

Was defined as the presence of hyperglycemia with fasting blood glucose >126 mg/dl; random blood glucose level >200 mg/dl; and/or glycated hemoglobin (HbA1c) >6.5%, with no previous history of diabetes, or of taking anti-diabetic medications, in patients admitted with COVID-19 infection.

### Inclusion criteria:

- All confirmed Covid-19 patients who had new-onset hyperglycemia, and were admitted to the COVID Complex of LRH, Peshawar.
- Aged 18-80 years.
- Either gender.

### Exclusion criteria:

- Pneumonia secondary to bacteria and other viruses like H1N1.
- All previously diagnosed diabetic patients.
- Pregnant patients (may have gestational diabetes).
- Patients with pancreatitis (risk of hyperglycemia).

The above-mentioned conditions may act as confounders and introduce bias in the study results. Prior approval from the Institutional Review Board (IRB) was taken for the data collection and research publication. Data of 300 confirmed COVID-19 patients with new onset hyperglycemia, who were admitted to the Department of Medicine/COVID Complex, Lady Reading Hospital, Peshawar were collected from the Health Management Information System (HMIS) of the hospital. It is one of the main hospitals of the Khyber Pakhtunkhwa province where most of the COVID-19 patients were admitted. History was obtained from the HMIS regarding demographic details, respiratory, and osmotic symptoms of polydipsia and polyuria. Examination findings were recorded including vitals like, pulse, blood pressure, temperature, respiratory rate, oxygen saturation, and systemic examination findings.

Results of investigations of blood samples were obtained including complete blood count, blood glucose level, Glycated hemoglobin (HbA1c), urea, creatinine, alanine aminotransferase; inflammatory markers like serum Ferritin, D-dimers and lactate dehydrogenase, and arterial blood gases (ABGs). **C**hest x-ray films were checked for the presence of infiltrates. Results of the nasopharyngeal swabs were obtained for Antigen based rapid detection test and Reverse transcription polymerase chain reaction (RT-PCR) assays.

### Statistical Analysis:

A predesigned proforma was utilized for recording the above-mentioned information. Data were entered and analyzed by SPSS version-23. Descriptive statistics were used to describe general information of the patients.

Continuous data like age, duration of symptoms, RBS, and HbA1c, were presented as mean ± standard deviation and range. Categorical data like sex, and the presence of new-onset diabetes mellitus, were presented as percentages and frequencies. The frequency of new-onset diabetes mellitus in patients with COVID-19 was determined. Data were stratified among age and gender and analyzed with a post-stratification chi-square test to see effect modification. The level of significance was set at p≤0.05. All results were presented in the form of tables and figure.

### Ethical approval:

The study was approved by the Institutional Review Board of Lady Reading Hospital, Peshawar, Pakistan, vide Ref: No. 335/LRH/MTI, dated: 25^th^ April 2022. The requirement for written informed consent from the patients was waived by the IRB, given the retrospective study design.

## RESULTS

Among the 300 COVID-19 patients, 163(54.3%) were female and 137(45.7%) were male. Forty-four (14.7%) patients had new-onset DM (NODM); 19(6.33%) were male and 25(8.33%) females (*p*=0.720), as shown in Table-I. The mean age of the patients was 56.80±13.72, (IQR=15) years. Other descriptive statistics are shown in Table-II.

**Table-I:** Gender distribution of NODM in COVID-19 patients (n=300).

Gender	No. of COVID-19 Patients		New onset Diabetes Mellitus	

	Frequency	Percentage	Frequency	Percentage
Male	137	45.7%	19	6.33%
Female	163	54.3%	25	8.33%
Total	300	100.0%	44	14.7%

Chi-Square p-value: X^2^(df1)=0.128, p=0.720.

**Table-II:** Descriptive Statistics of COVID-19 Patients (n=300).

Variables	Minimum	Maximum	Mean	Standard Deviation
Age	16	100	56.80	13.72
Symptom duration in days	02	12	13.30	12.66
Blood Glucose Level	95	365	142.88	53.17
Hb. A1c* Level	4.4	12.8	05.83	01.35

* Glycated hemoglobin A1c.

**Fig.1:**
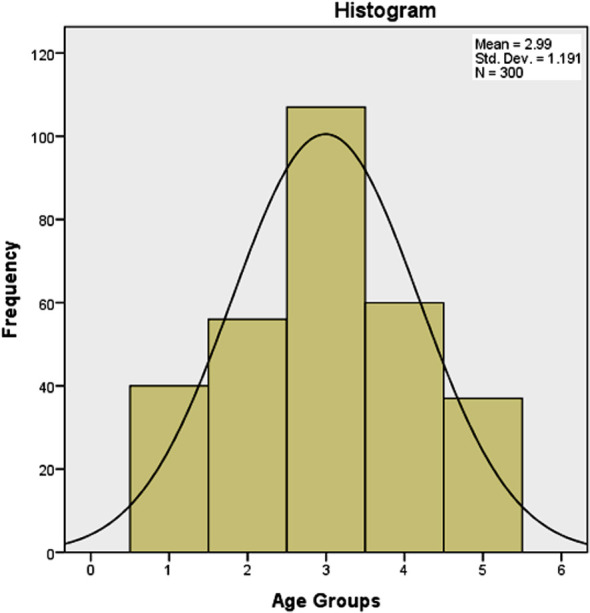
Age group distribution of COVID-19 patients (n=300).

The majority (54.4%) of the COVID-19 patients were between 41 to 60 years age, as depicted in Fig.1, showing a normal distribution pattern. Age-wise distribution of the NODM patients is shown in Table-III, revealing that most of the patients with NODM were of the middle age (*p*=0.018).

**Table-III:** New-onset Diabetes in different age groups of COVID-19 Patients (44/300).

Age groups	New onset Diabetes Mellitus	

	Frequency	Percentage
16-40	02	0.7%
41-50	14	4.7%
51-60	14	4.7%
61-70	12	4.0%
Above 70	02	0.7%
Total	44	14.7%

Pearson Chi-Square p-value: X^2^(df 4)=11.878, p=0.018.

## DISCUSSION

The mean age of our study patients was 56.80±13.72 years, dominated by women (54.3%). Among the 44 NODM patients, the majority (57%) were female (*p*=0.720). Similar findings have been reported from Wuhan, China^7^, and Peshawar^11^, supporting our study results. On the other hand, predominantly male patients (67%) were reported from India.^12^

We recorded NODM in 44(14.7%) of the COVID-19 patients. Studies from around the world have reported a widely varying proportion of newly diagnosed diabetes, from 0.6% to 46.2% in severe or critically ill COVID-19 patients.^8^ Our study results were corroborated by many Indian studies. Kreethi et al.**12** in Telangana, India found NODM in 15% of COVID-19 patients in a tertiary care hospital. In Chennai^13^, India, a study reported an incidence of 20.6% in hospitalized COVID19 patients. Similarly, Sathish et al.^8^, in a meta-analysis of eight studies conducted in the United States of America, Italy, and China reported a prevalence of NODM in 14.4% of the 3711 patients. Another systematic review of 27 studies of 3241 patients with a mean age of 43.21±21.0 years revealed NODM in 16.1%; 9.74% in the developed world and 19.5% in the developing world. The overall mortality in NODM patients was 14.5%.^9^

However, on the other hand, Sane et al. reported 31.1% NODM in hospitalized COVID19 patients in Addis Ababa, Ethiopia.^14^ In a similar study in Germany, on 35865 patients, Rathmann et al.**15** found an increased risk of diabetes in COVID19 patients with mild to moderate disease. Fagarasan et al.^16^ from Romania also reported NODM in 26.48% of COVID-19 patients. In Aswan, Egypt, 28.5% incidence of NODM in COVID-19 patients was reported by Sayed et al.^17^ Pre-existing diabetes in COVID-19 carries a worse prognosis and so is the case with new-onset diabetes in moderate to severe hospitalized patients.^2^,^10^ Several mechanisms have been described by which COVID-19 infection increases the chances of NODM and worsening of the pre-existing diabetes.^2^-^4^,^6^,^18^ The COVID-19 induced NODM is thought to be a novel form of DM, with higher mortality and worse prognosis compared to those with pre-existing DM.^2^-^4^,^5^,^19^ COVID-19 infection in people with diabetes, including NODM, can cause marked hyperglycemia through several complex but interrelated factors.^3^,^4^,^6^,^20^ SARS-CoV-2 may enter the pancreas and beta cells via the expression of angiotensin-converting enzyme-2 (ACE-2) receptors.

The virus impairs pancreatic insulin secretion by inflammation, hypoxia, and injury to the beta cells thereby either aggravating DM or triggering new-onset DM. Infection with the virus itself leads to oxidative stress, resulting in hypoxia and inflammation, which aggravates glucose homeostasis.^4^,^7^ Another underlying mechanism appears to be insulin resistance due to the high levels of interleukin-6 (IL-6) and tumor necrosis factor-alpha (TNF-a) in subjects with severe COVID-19 infection.^21^-^23^ Additionally, damage to key organs involved in glucose metabolisms, such as the kidney and the liver, resulting in abnormal blood glucose levels have been observed in cases of COVID-19 infection.

The use of corticosteroids is common in these patients, which also contributes to hyperglycemia. Metabolic inflammation caused by high blood glucose levels affects the body’s immune system and the process of healing thereby prolonging recovery.^7^,^21^,^22^ Hyperglycemia has been found to affect lung volume, causes down-regulation of ACE-2 receptors, which has a protective effect against inflammation, and in turn leads to inflammatory damage by the virus and potential cytokine storm leading to increase morbidity and mortality.^21^-^23^

### Study Findings and Practice Implications:

New-onset diabetes during or after acute COVID-19 infection is an important new public health problem. Being first-line physicians involved in the management of COVID-19 patients; we have found patients of different age groups developing this new phenomenon of NODM. On follow up, some of these NODM patients recovered completely from NODM and became normoglycemic even without anti-diabetic medications. Another significant observation was the increased amount of insulin required to control the hyperglycemia in these COVID-19 patients compared to non-COVID-19 patients with DM.

Patients with diabetes and hyperglycemia have an increased risk of severe infection, complications and mortality. COVID-19 patients with newly diagnosed diabetes are more likely to die than those with known diabetes. This study is important to know the frequency of NODM in patients with severe COVID-19 infection admitted to the hospital so that they are managed early to reduce mortality. Patients must be regularly monitored for NODM so that early therapy is started to control hyperglycemia and improve outcome.

### Limitations:

The present study includes a comparatively small sample size, a single-center study, and a retrospective design. Further large scale, multicenter studies are needed to explore the exact causative association of the Coronavirus with the development of new-onset diabetes mellitus in patients suffering from acute COVID-19 infection

## CONCLUSION

In conclusion, a significant number of patients with COVID-19 are prone to develop new-onset diabetes during their admission to the hospital which carries a poor prognosis as compared to the non-diabetics.

### Authors Contribution:

**MARA:** Conceived the idea, designed the study, manuscript writing/editing and final approval.

**ZA**, **NI**, & **UZ:** Did data collection; statistical analysis & manuscript review/editing and final approval.

**NI** is responsible and accountable for the accuracy/ integrity of the work.
